# A New Super Resolution Framework Based on Multi-Task Learning for Remote Sensing Images

**DOI:** 10.3390/s21051743

**Published:** 2021-03-03

**Authors:** Li Yan, Kun Chang

**Affiliations:** School of Geodesy and Geomatics, Wuhan University, 129 Luoyu Road, Wuhan 430079, China; lyan@sgg.whu.edu.cn

**Keywords:** multi-task learning, Gaussian blur kernels, convolutional neural network, unsupervised learning strategy

## Abstract

Super-resolution (SR) algorithms based on deep learning have dominated in various tasks, including medical imaging, street view surveillance and face recognition. In the remote sensing field, most of the current SR methods utilize the low-resolution (LR) images that directly bicubic downsampled the high-resolution (HR) images as not only train set but also test set, thus achieving high PSNR/SSIM scores but showing performance drop in application because the degradation model in remote sensing images is subjected to Gaussian blur with unknown parameters. Inspired by multi-task learning strategy, we propose a multiple-blur-kernel super-resolution framework (MSF), in which a multiple-blur-kernel learning module (MLM) optimizes the parameters of the network transferable and sensitive for SR procedures with different blur kernels. Besides, to simultaneously exploit the prior of the large-scale remote sensing images and recurrent information in a single test image, a class-feature capture module (CCM) and an unsupervised learning module (ULM) are leveraged in our framework. Extensive experiments show that our framework outperforms the current state-of-the-art SR algorithms in remotely sensed imagery SR with unknown Gaussian blur kernel.

## 1. Introduction

In digital image processing, low-resolution (LR) images are generally viewed as a result of a degradation function of high-resolution (HR) images. Although the degradation functions of real-world LR-HR image pairs have infinite expressions and parameters, which makes the super-resolution (SR) task an ill-posed problem, the SR algorithm aims to find a relatively simplified degradation model and effectively enhance the resolution of LR images.

In the remote sensing field, HR images provide strong data support to a set of essential tasks, such as disaster monitoring, national resource management, and weather prediction. However, due to the limitation of imaging devices, most present databases consist of low-resolution images rather than high-resolution ones. Therefore, SR algorithms for optical remote sensing images have been a hot topic for decades. There are two widely explored SR methods: single-image SR (SISR) and multi-image SR (MISR). MISR utilizes multiple images of the same scene to reconstruct a single image, which fully exploits extra information to synthesize the complete scene. Intuitively, MISR should have played a significant role in SR for remotely sensed imagery because cameras in satellites, airplanes and drones periodically generate images of a same scene. Nevertheless, the problems, such as image alignment, climate variation, and the change of scene content, inhibit the MISR application in remote sensing images. In contrast, SISR, in conjunction with the deep learning architecture based on Convolutional Neural Networks (CNN) and Generative Adversarial Networks (GAN), shows remarkable potential. Currently, the SISR algorithms that achieve state-of-the-art performance often involve advancement in deep learning.

ESPCN [1] achieved state-of-the-art performance in SR tasks by introducing spatio-temporal sub-pixel convolution networks that made full use of temporal redundancies and maintained an excellent balance between reconstruction accuracy and running time. SRFBN [2] added deconvolution layers to generate residual images in the feedback block and achieved state-of-the-art performance in DIV2K [3]. DRLN [4] allowed the low-frequency information to focus on the high-level features by employing cascading residual on the residual structure. Through adding a recursive block that makes up of multiple residual units and sharing weights across all these units, DRRN [5] achieved the state-of-the-art performance in SR task with fewer parameters (300 K) than other SotA architectures for SR (i.e., DRCN [6] 1.8 M). SAN [7] extracted large-range spatial contextual information by exploiting the second-order feature statistics. Besides, the SR algorithms based on deep learning have been also applied into the SR task in remote sensing field. Lanaras et al. [8] employed a CNN architecture to upsample the LR images in an end-to-end manner to super-resolve the multi-spectral imagery delivered by the Sentinel-2 satellite mission from about 60 Ground Sampling Distance (GSD) to 10 GSD. Shen et al. [9] proposed a residual convolutional neural network in order to generate HR PolSAR images from LR ones, which focused on the change of pixel-wise difference instead of the slight but complex transformation between corresponding pixels. Salvetti et al. [10] proposed a fully convolutional residual attention multi-image super-resolution (RAMS) to exploit spatial and temporal correlations to leverage multiple remote sensing images.

Although deep learning algorithms have shown tremendous success in SR tasks, some problems still exist. In many studies [11,12,13], the LR images in both train set and test set are obtained by bicubic downsampling HR images, thereby making these networks have high PSNR/SSIM scores on the test set but show performance drop in real scenarios (i.e., remote sensing images). In other words, the bicubic downsampling method, unlike the degradation model of the real scene, is over-simplified so that the test on bicubic downsampled LR image cannot precisely represent the generalization ability of the networks. Moreover, in real scenarios, each LR-HR pair has a different degradation function and parameters. Conventional data augmentation methods (e.g., synthesizing data through various degradation function) not only increase computation expense but also cannot achieve satisfying performance on every test image. Besides, designing deeper networks, despite the better performance, is likely to result in vanishing gradient problems and exploding gradient problems.

To address these issues, we propose a multiple-blur-kernel super-resolution framework (MSF), which utilizes external and internal information simultaneously. Specifically, inspired by the multi-task learning strategy, we present a multiple-blur-kernel learning module (MLM) to wisely learn the general features among SR with different blur kernels. In addition, a class-feature capture module (CCM) is embedded into the framework in advance, learning the powerful image prior from the large-scale database and accelerating the training process in MLM. Moreover, the predictive power from internal image statistics in a single remote sensing image can further promote the performance of the framework. Therefore, an unsupervised learning module (ULM) follows the MLM, exploiting the data repetition in the test image. In general, our contributions are summarized as follows:

(1) We design a simplified but effective CNN architecture with residual learning as the foundation network. It focuses on the slight difference between LR-HR image pairs and remarkably decreases the training cost because the residuals in most region are close to zero. Moreover, a Gaussian blur kernel generator is introduced to randomly generate both isotropic and anisotropic Gaussian blur kernel, simulating the SR process in real world instead of the predefined upgrade model.
(2) We propose a multiple-blur-kernel learning module based on the multi-task learning strategy, in which each task corresponds to a randomly generated Gaussian blur kernel. The multi-task learning strategy forces the network to pay attention to general features among LR-HR image pairs with different Gaussian blur kernels, considerably boosting the SR performance when testing the image pair with unknown blur kernel. Besides, through learning large-scale dataset, the natural priors from the remote sensing images provide useful class-specific edge and texture information for SR and accelerate the training process in MLM. In addition, the unsupervised learning module further improves the SR performance by exploiting the recurrent information inside the test image.
(3) We test the performance of MSF and other SotA SR architectures on the various kinds of instances in remote sensing images that degrade with a randomly generated Gaussian blur kernel. Then we study the influence of different scaling factors on these SR architectures. Subsequently, we conduct the ablation experiment to test the effectiveness of three modules. Finally, we test our MSF’s performance on real-world images without the ground truth. Extensive experiments show that the performance of our framework outperforms other SR architectures on remote sensing images with unknown blur kernels.

## 2. Method and Material

### 2.1. Proposed Method

In digital image processing, the relationship between the HR image and LR image is generally abstracted into the degradation model:

(1) $$I_{\mathrm{LR}}^{k}=\left(I_{\mathrm{HR}}*k\right)↓s+n$$

where $I_{\mathrm{LR}}^{k}$, $I_{\mathrm{HR}}$, *, *k*, ${↓}_{s}$ and n denote the LR image, HR image, convolution, blur kernel, downsampling with scaling factor *s* and noise, respectively.

In the photogrammetry field, a large number of factors (e.g., sensor errors, climate, and data compression) have an effect on the imaging process. Therefore, the blur kernels *k* in Equation (1) are usually treated as Gaussian blur kernels. The commonly used method that generates the train set is to convolute the HR image with a specific Gaussian blur kernel and then perform the bicubic downsampling step. When the test set is generated by the same Gaussian blur kernel, the SR framework show excellent result. However, a deep learning network training on images with a specific Gaussian blur kernel often shows performance drop on the test set with different Gaussian blur kernel.

Theoretically, the data augmentation method that synthesizes the LR-HR pairs by various Gaussian blur kernels can enhance the performance of the SR model when facing the test set with unknown Gaussian blur kernel. But in most cases, the process of learning the expanded dataset considerably consumes the computational resources, along with gradient vanishing problems and gradient exploding problems. Hence, we introduce the multi-task learning strategy that can wisely learn the relationship among SR process with different blur kernels. Multi-task learning refers to a learning strategy that the network captures the domain-specific knowledge in multiple related tasks (e.g., semantic segmentation and object detection) in order to promote the generalization ability across these tasks. We propose a framework in which every task corresponds to a SR process with a specific blur kernel, aiming to learn the general feature among multiple SR processes with different blur kernels. Meanwhile, we exploit the usefulness of both external instance prior information and internal recurrent information to further promote the SR result.

**Figure 1:**
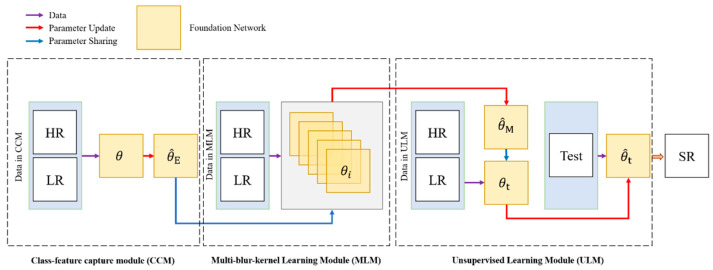
The overview of the Multiple-blur-kernel Super-resolution Framework (MSF).

The overview of our proposed Multiple-blur-kernel Super-resolution Framework (MSF) is shown in Figure 1. There are three modules in our MSF: the class-feature capture module (CCM) extracts representative priors (texture and edge) of corresponding instances (plane, ship, or car) in remote sensing images through learning large-scale external dataset, the multiple-blur-kernel learning module (MLM) initializes the parameters by these priors and intends to find a point in the parameter space transferable and sensitive for various gradient descent zone for different blur kennels, and the unsupervised learning module (ULM) combines internally recurred information in the entire test image to perform the blur kernel estimation.

**Figure 2:**
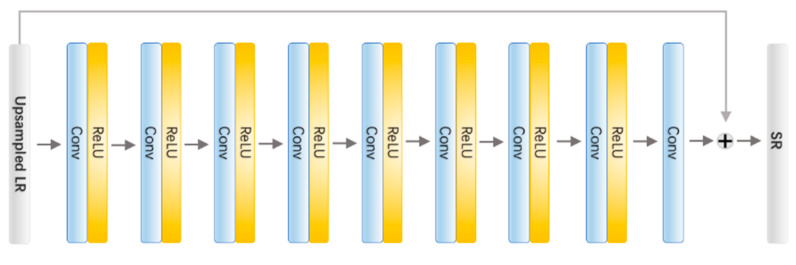
The architecture of the foundation network. The network takes upsampled LR image as input tensor with shape H×W×C×F, where H, W, C and F denote height, width, channels (3 as default) and the number of filters (64 in our setting), respectively.

The core of the multi-task learning strategy is the parameter sharing mechanism, which requires all modules in the framework to rely on the same foundation network. As is shown in Figure 2, we employ a simple convolutional network with residual learning as the foundation network. The foundation network consists of 8 hidden convolutional layers. Each of the first 7 convolutional layers has 64 filters and is activated by ReLU. The last convolutional layer has 3 filters, and the output of this layer is connected with the original input in order to force the network to focus on the pixel-wise difference between the LR-HR image pair.

#### 2.1.1. Class-Feature Capture Module (CCM)

A multi-task learning strategy consumes considerable computational resources because parameter updating in MLM needs to leverage the losses of all subordinate networks. Theoretically, the more subordinate networks in MLM, the better performance the framework achieves on the test set with unknown blur kernels. However, adopting too many subordinate networks enlarges the size of the framework and brings various difficulties in the training process. In practice, the video memory of GPU constrains the number of subordinate networks, thus limiting the performance of the entire framework. Therefore, some optimization steps that decrease the computation burden are needed. It is shown that the class-feature from large-scale external dataset can provide significant priors of natural scenarios (e.g., texture and edge of the instances). The initialization with these priors, as opposed to the random initialization, reduces the number of iterations, and thus accelerates the training phase in MLM. We propose a network whose architecture is the same as the network in MLM. The network learns instance-relevant representative features by training on the external dataset. The optimized parameter in CCM will be shared with networks in MLM. The details in CCM are shown below.

Through bicubic downsampling the HR images (whose class is the same as the test piece), we obtain the LR-HR pairs (denoted as $D\sim \left(I_{\mathrm{HR}},I_{\mathrm{LR}}^{\mathrm{bic}}\right)$) for the class-feature capture module (CCM), in which the LR images are the input of network and the HR images are ground truth. The network is randomly initialized and is optimized with the pixel-wise L1 loss:

(2) $$Loss^{D}\left(\theta \right)=E_{D\sim \left(I_{\mathrm{HR}},I_{\mathrm{LR}}^{\mathrm{bic}}\right)}\left[∥I_{\mathrm{HR}}-f_{\theta }\left(I_{\mathrm{LR}}^{\mathrm{bic}}\right){∥}_{1}\right]$$

(3) $$\theta_{E}\leftarrow \theta$$

After the training process in CCM, the optimized parameter $\theta_{E}$ represents the implicitly class-relevant features across the large-scale data and is saved to initialize all networks in MLM.

#### 2.1.2. Multi-Blur-Kernel Learning Module (MLM)

**Figure 3:**
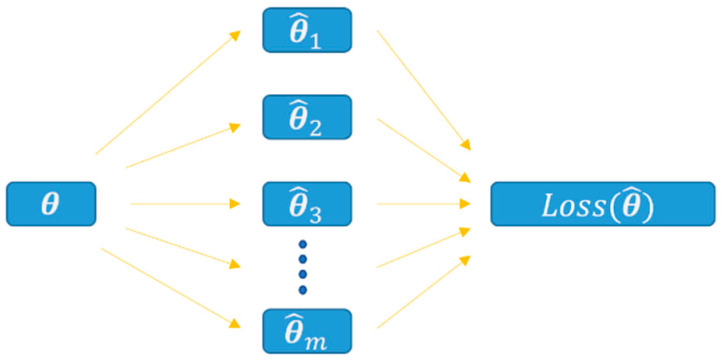
Diagram of the new loss function that leverage all subordinate losses, where θ denotes the parameter of the main network and $\theta_{1},\;\theta_{2},\theta_{3},\ldots ,\theta_{m}$ denote the parameters of the subordinate networks.

The core of the multi-task learning strategy is the parameter sharing mechanism. Two methods are commonly used: hard sharing and soft sharing. To overcome the gradient vanishing problems brought by hard sharing and considerable training expense from soft sharing, we propose a joint sharing mechanism. A new loss function is devised to leverage all loss of the multiple tasks (i.e., parameter optimization for multiple blur kernels $\theta_{1},\;\theta_{2},\theta_{3},\ldots ,\theta_{m}$ in Figure 3). Mathematically, the MLM serves the function of finding a point in the parameter space sensitive enough to perform gradient descent to different local minimum.

The number of subordinate networks is a hyperparameter. Theoretically, MLM with larger number of subordinate networks has better generalization ability. Nevertheless, the algorithmic complexity of the MLM is O($n^{2}$). As a result, there is a tradeoff between efficiency and effectiveness.

After determining the number of subordinate networks, the train set in MLM needs to be prepared before the learning phase. First, we need some blur kernels. To randomly generate Gaussian blur kernels, we design a Gaussian blur kernel generator based on a covariance Σ:

(4) $$\Sigma ={M\Lambda M}^{t}$$

(5) $$\Lambda =\left(\begin{array}{cc} \lambda_{1} & 0\\ 0 & \lambda_{2} \end{array}\right)\lambda_{1}\sim U\left(1,\;2s\right)\lambda_{2}\sim U\left(1,\;\lambda_{1}\right)$$

(6) $$Μ=\left(\begin{array}{cc} \cos\varphi & -\sin\varphi \\ \sin\varphi & \cos\varphi \end{array}\right)\;\varphi \sim U\left(0,\;\pi \right)$$

where φ denotes random angle, $\lambda_{1}$ $\lambda_{2}$ denote random length in two axes, and *s* denotes the scaling factor. Note that when $\lambda_{1}=\lambda_{2}$ and φ = 0, the generated Gaussian blur kernel is isotropic Gaussian blur kernel; otherwise it is anisotropic Gaussian blur kernel.

After generating *m* Gaussian blur kernels, we randomly select *m* sets of HR remote sensing images. For each set of data, we downsample the HR images to generate the LR counterparts by using a corresponding blur kernel. Then the LR-HR pairs are divided into two groups, the training data $D_{\mathrm{train}}^{i}$ and validation data $D_{\mathrm{val}}^{i}$ (*i* denotes the *i*th blur kernel)

The architecture of both main and subordinate networks is the foundation network, and every network in MLM initializes with the parameters $\theta_{E}$. The parameter update in MLM is a loop. First, each subordinate network trains on $D_{\mathrm{train}}^{i}$ and performs a or a few steps of gradient descent. The parameter update for one gradient descent is:

(7) $$\theta_{j+1}^{i}\leftarrow \theta_{j}^{i}-\alpha \nabla_{\theta }Loss^{D_{\mathrm{train}}^{i}}\left(\theta \right)$$

where $Loss^{D_{\mathrm{train}}^{i}}$ is the pixel-wise L1 loss, *j* is the number of gradient descent steps, and α is the learning rate of the gradient descend on trainset (0.0001 in our experiment). Normally the value of gradient descent steps in subordinate networks ranges from 1 to 10, because too many steps of gradient descent hinder the generalization ability of MLM. We set *j* = 3 as default. Second, the validation set $D_{\mathrm{val}}^{i}$ is sent to updated network and calculate the L1 loss $Loss^{D_{\mathrm{val}}^{i}}\left(\theta_{j}^{i}\right)$. Third, on the basis of $Loss^{D_{\mathrm{val}}^{i}}\left(\theta_{j}^{i}\right)$, the main network uses a new objective function to optimize its parameter:

(8) $$\arg\min\;\overset{m}{\underset{i=1}{\sum }}Loss^{D_{\mathrm{val}}^{i}}\left(\theta_{j}^{i}\right)$$

(9) $$=\arg\min\;\overset{m}{\underset{i=1}{\sum }}Loss^{D_{\mathrm{val}}^{i}}\left(\theta -\alpha \nabla_{\theta }Loss^{D_{\mathrm{train}}^{i}}\left(\theta \right)\right)$$

The parameter update for one gradient descent in main network is shown as follows:

(10) $$\theta \leftarrow \theta -\beta \nabla_{\theta }\overset{m}{\underset{i=1}{\sum }}Loss^{D_{\mathrm{val}}^{i}}\left(\theta_{j}^{i}\right)$$

where β is the learning rate of the main network (0.001 in our experiment).

Finally, the updated parameter in main network is copied to every subordinate network, and next loop begins. The train process ends till the main network converges.With the parameter optimization from MLM, the ULM that takes the small piece containing the interesting instance as input can efficiently descend to the local optimum.

Algorithm 1 shows the training steps in MLM:
**Algorithm 1. Training process in a multiple-blur-kernel learning module**Input: The number of subordinate networks (blur kernels), randomly selected HR images, the optimized parameter $\theta_{E}$ from CCM, the learning rate α
βOutput: parameter $\theta_{M}$Generate m Gaussian blur kernels **For** all Gaussian blur kernels **do** randomly select HR images downsample the HR images with corresponding Gaussian blur kernel divide the HR-LR pairs into $D_{\mathrm{train}}$ and $D_{\mathrm{val}}$ **End** Initialize parameters of all networks in MLM with $\theta_{E}$ **While** the main network does not converge **do** **For** all subordinate networks **do** **For** j steps **do** Update the parameter by Equation (7) **End** Evaluate the loss in validation set $Loss^{D_{\mathrm{val}}}\left(\theta \right)$ **End** Update the parameter in main network by Equation (10) Copy θ to all subordinate networks **End**

The methodology and structure of MLM is similar to MAML [14] both of which aim to promote the generalization ability of the framework, but the details of two frameworks are quite different. the MAML focuses on sampling not only from the data but also the task whereas the multi-task learning strategy pay attention to the common features among tasks, which, in our work, are the features among various SR processes with different Gaussian blur kernels. In MLM, samples of same instances are randomly selected to form some groups and then degraded through different blur kernels. Conversely, MAML requires the task distribution before the training process.

#### 2.1.3. Unsupervised Learning Module (ULM)

Although our proposed MLM can remarkably perform SR with unknown blur kernels, kernel estimation for the test set can further promote the performance of the SR framework. The underlying blur kernel of a single image can be predicted by using the self-similarity properties within the image [15], which is usually learned by unsupervised learning.

In a single remote sensing image, small pieces of information (e.g., object, texture, patch, edge) recurs in different scales, which is a significant property for super resolution. Aided by the cross-scale internal recurrence of a single remote sensing image, we seek to learn the degradation relationship between the specific image and its downsampled counterpart. We use an unsupervised strategy that depends on two concerns:

(1) The upgrade function between the SR image and test image (LR) is similar to that between the test image (LR) and its downsampled counterpart. The upgrade function is denoted as *F*:

(11) $$I_{\mathrm{LR}}=F_{1}\left({\left(I_{\mathrm{LR}}\right)}_{\mathrm{downsample}}\right)$$

(12) $$I_{SR}=F_{2}\left(I_{LR}\right)$$

(13) $$F_{1}\approx F_{2}$$

(2) The small pieces within a single remote sensing image obey the same SR model (i.e., same Gaussian blur kernel). Therefore, we divide the test image into several small parts and send them into the network in ULM to estimate the blur kernel.

**Figure 4:**
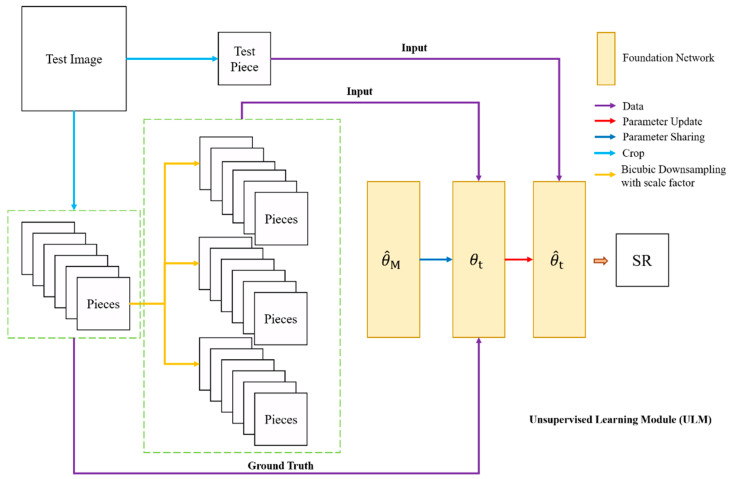
The framework of the unsupervised learning module (ULM).

As shown in Figure 4, the purpose of ULM is to fully exploit the recurred textures and edges in a single remote sensing image to estimate the blur kernel of the test image through using the unsupervised learning strategy. Based on the concern that the SR procedures of all pieces within a single remote sensing image encounter the same degradation function (i.e., Gaussian blur kernel with same parameter), we divide the test image into multiple pieces. Each piece that arises from the test image has the same size with a slight section overlapped because the size of the image may be not divisible by the size of the input tensor of the foundation network. This overlap setting makes our ULM tackle any size of test image, and the final SR result can be reconstructed by multiple outputs of the network in ULM. It should be noted that too many input pieces generated from test images weaken the networks ability to super resolve the complete instance because the reconstruct step is lack of semantic correlation, although this step contributes to promoting the contrast and sharpening the edges in the result.

With the multiple pieces from the test image, we then generate downsampled version of these piece. As shown in Equations (11)–(13), our learning strategy in ULM depends on the concern that the SR procedure from downsampled version of the test piece to the original test piece is similar to that from test piece to SR results. Consequently, we resize the test pieces into multiple small versions (scaling factors 0.9, 0.8, 0.7, 0.6, 0.5), and then bicubic interpolate them back into the original size, synthesizing multiple LR-HR image pairs. The aim of this step is to let the network focus on general structural information of these test pieces.

There are only two foundation networks in the ULM. The first network initializes with $\theta_{M}$, takes all pieces that are generated from test image as input tensor, and optimizes with L1 loss, as mentioned in Equation (2). Once the first network coverages, the second network shares the optimized parameter, and the piece containing the desired instance is sent into the second foundation framework as test input. More results are obtained by data augmentation (i.e., rotation by 90, 180, 270 degrees, and reflection both vertical and horizontal). The final SR result is synthesized from the intermediate results by using the stacking step. We employ median rather than mean of all predicted outputs as the final result because the median value can maintain the sharpen contrast within the image whereas the mean value usually smooths the variation, generating a relatively blurred result.

Since the CCM and MLM fully utilize the external information and optimize the parameters to a point that can efficiently descend, the USM only needs one or a few iterations to show outstanding performance. Due to parameter optimization in MLM, the time and computational consumption of ULM noticeably decreases. Contrary to some unsupervised SR models [15,16], which commonly requires more than 10K times of gradient update, the ULM needs only a few steps of gradient update (normally less than 10) to achieve excellent performance on the test image. (all abbreviations of terms are shown in section of Abbreviations).

### 2.2. Dataset

To comprehensively evaluate the performance of our proposed MSF, we exploit the DIOR dataset [17]. The DIOR dataset is one of the largest publically available object detection datasets in the remote sensing field. In DIOR, there are 23,463 optical remote sensing images and 192,472 object instances labeled with 20 categories. The spatial resolution of images varies from 0.5 m to 30 m. This large range of spatial resolution variation dramatically promotes the effectiveness of our MSF on images with different spatial resolutions. In addition, the object instances extracted from the remote sensing images are taken as the input of CCM, providing strong natural image priors to improve the SR performance of MSF on the test piece containing the same class of instance. What is more, the size of the image in DIOR is 800 × 800 pixels, large enough to be divided into a number of pieces (256 pieces in our experiment) to perform blur kernel estimation in the test step. We do experiments on five categories of object instance: plane, ship, vehicle, stadium and storage-tank. The dataset can be downloaded from (https://pan.baidu.com/share/init?surl=w8iq2WvgXORb3ZEGtmRGOw passcode: 554e; accessed date: 2 March 2021).

### 2.3. The Evaluation Indexes

To evaluate the performance of the SR algorithm, some image quality assessment methods (IQA) are proposed. There are two types of IQA methods: subjective methods, which mainly depend on the human visual perception, and objective computational methods, which focus on the pixel-wise difference and similarity between LR and HR pairs. In remote sensing field, although subjective indexes are able to accurately capture the human visual attention, objective indexes are more commonly used to obtain quantitative result.

Peak Signal-to-Noise Ratio (PSNR) is a measurement that widely assesses the loss transformation in image processing tasks, such as image compression and image inpainting. For SR task, PSNR is defined as follows:

(14) $$\mathrm{PSNR}=20\cdot \log_{10}\left(\frac{L^{2}}{\frac{1}{N}\sum_{i=1}^{N}{\left(I\left(i\right)-\hat{I}\left(i\right)\right)}^{2}}\right)$$

where *L* denotes the maximum pixel value (i.e., equals to 255 in general cases using 8-bit representations). I and $\hat{I}$ denote the ground truth image and predicted image, respectively.

Another widely used index is the structural similarity index (SSIM). In the human brain, structural information provides more visual stimuli. On the basis of this scientific observation, SSIM is proposed for measuring the structural similarity between images, based on independent comparisons in terms of luminance, contrast, and structures. For an image, the comparison of luminance and contrast, denoted as $C_{l}\left(I,\hat{I}\right)$ and $C_{c}\left(I,\hat{I}\right)$, are estimated by the mean and standard deviation the image intensity, and are obtained by following equations:

(15) $$C_{l}\left(I,\hat{I}\right)=\frac{2\mu_{I}\mu_{\hat{I}}+C_{1}}{\mu_{I}{}^{2}+\mu_{\hat{I}}{}^{2}+C_{1}}$$

(16) $$C_{c}\left(I,\hat{I}\right)=\frac{2\sigma_{I}\sigma_{\hat{I}}+C_{2}}{\sigma_{I}{}^{2}+\sigma_{\hat{I}}{}^{2}+C_{2}}$$

where $C_{1}$ and $C_{2}$ are constants for stability:

(17) $$C_{s}\left(I,\hat{I}\right)=\frac{\sigma_{I\hat{I}}+C_{3}}{\sigma_{I}\sigma_{\hat{I}}+C_{3}}$$

(18) $$\mathrm{SSIM}\left(I,\hat{I}\right)={\left[\;C_{l}\left(I,\hat{I}\right)\right]}^{\alpha }{\left[C_{c}\left(I,\hat{I}\right)\right]}^{\beta }{\left[\;C_{s}\left(I,\hat{I}\right)\right]}^{\gamma }$$

where α, β, γ are control parameters for adjusting the relative importance.

## 3. Experiments and Results

### 3.1. Data Preparation

Some pre-processing steps need to be done before running the framework:

#### 3.1.1. Bicubic Downsampling Step

Bicubic downsampling is a widely adopted step in SR models because the equal size of the input LR images and the ground truth forces the SR models focus on the slight transformation between corresponding pixels among LR-HR image pair, decreasing the complexity of the models and increasing the generalization ability:

- direct downsample with scaling factor;
- bicubic interpolate back into the origin size.

All networks in MSF take pieces (64 × 64 pixels) as input tensor.

#### 3.1.2. Data Preparation in CCM

The purpose of CCM is to exploit the prior information in large-scale external dataset. Due to the diverse spatial resolutions of observation instances, CCM can extract strong predictive information (i.e., edges and textures) to SR procedure. The class-name information can be read from the annotation document. Besides, instances out of size (larger than 64 × 64 pixels) can be cropped to the size needed. It is shown that the pieces containing incomplete instance as external data do not hinder the performance of CCM. Instead, these pieces usually contain high spatial resolution object, providing useful textures and edges to test objects with low spatial resolution:

- randomly select 200 images and crop small pieces (64 × 64 pixels) containing instance. The pieces work as external dataset;
- bicubicly downsample the pieces in external dataset, and synthesize the paired dataset $D_{E}$.

#### 3.1.3. Data Preparation in MLM

MLM serves the function of letting the framework capture the implicit generalization features among different Gaussian blur kernels. Therefore, the intuitive data pre-processing step is to synthesize data from $D_{E}$ with generated Gaussian blur kernels. However, it is proved by the experiments that the optimization from newly selected instance-pieces excels this intuitive data-augmentation step. So we select new images containing same-class instance as train data in MLM. Moreover, although more blur kernels and more data for each subordinate network theoretically enhance the performance of MLM, the algorithm complexity of MLM is O($n^{2}$). It is a tradeoff between computational cost and effectiveness:

- randomly select 160 images of the same class and crop small pieces (64 × 64 pixels) containing instances;
- randomly generate 16 Gaussian blur kernels;
- every 10 pieces bicubicly downsample with a Gaussian blur kernel, 8 pieces work as $D_{\mathrm{train}}$, 2 pieces work as $D_{\mathrm{val}}$.

Bicubic downsampling with a Gaussian blur kernel involves a blur convolution step between downsampling and bicubic interpolation.

#### 3.1.4. Data Preparation in ULM

The unsupervised learning strategy in ULM aims to perform a kernel estimation within the test piece, forcing the network to descend in a correct gradient descent zone and finding the local minimum for corresponding blur kernel. To solve the size of the image cannot be divisible by the size of input piece, we cut the test image into 256 pieces with a small part overlapped. For overlapped part, we reconstruct the SR piece through median value of the results from multiple pieces:

- divide the test image (800 × 800 pixels) into 256 pieces (64 × 64 pixels), some of which are slightly overlapped;
- for each piece, downsample by several scaling factors and then bicubic interpolate back to origin size (64 × 64 pixels), synthesizing the paired dataset;
- perform data augmentation including rotation (90,180,270 degrees) and reflection (horizontal and vertical).

The whole test set contains 100 randomly selected image-pieces with instances. As opposed to many studies that use bicubic downsampling images as the test set, we generate a new Gaussian blur kernel to perform convolution between the downsampling step and the interpolation step in the test images. Our purpose is to test the ability of MSF and other algorithms to super resolve images with unknown Gaussian blur kernel. We implement all experiments on a workstation equipped with an Intel CPU i7 8700 k, a RTX2080Ti GPU with 11 GB memory and 64 GB DDR4 Memory. Our framework is running on the Tensorflow framework version 1.8 and CDUA10.

### 3.2. Experiment and Results

#### 3.2.1. The Comparison with Other State-of-the-Art Super Resolution Algorithms

To comprehensively evaluate the effectiveness of our proposed method, several current state-of-the-art super-resolution approaches, including the conventional bicubic interpolation (i.e., LR input for MSF), EDSR [13], RCAN [18], and SRFBN [2], were compared. The table below shows the comparison of PSNR and SSIM metrics with these SotA methods on the test set. All algorithms are tested on the test set with a newly generated Gaussian blur kernel.

As shown in Table 1, our MSF shows excellent performance dramatically superior to all other SotA algorithms on nearly all classes of the test set expect on the stadium-class data (slightly inferior to SRFBN). We observe that it generally follows the order: MSF, SRFBN, RCAN, EDSR. In details, the PSNR/SSIM scores of EDSR and RCAN are only slightly higher than that of the direct bicubic-interpolation method, while SRFBN and our MSF show noticeably better results. Our MSF achieves obviously higher scores on the test sets of vehicle, ship, and plane classes (PSNR 6.35, 4.72 and 1.48 dB; SSIM 0.362, 0.176, 0.49, compared with the second-best results), but similar scores on storagetank class (PSNR 0.12 dB; SSIM 0.036). Our MSF also shows performance drop on stadium scenario, decreased by approximate 9 dB compared with performance on other four classes. Interestingly, the EDSR achieves inferior results compared with bicubic interpolation method on the stadium and storagetank classes, which suggests that EDSR cannot provide high-frequent information when testing these two scenes. We speculate the relatively inferior performance of EDSR results from its removal of the short-skip modules in the residual network.

**Figure 5:**
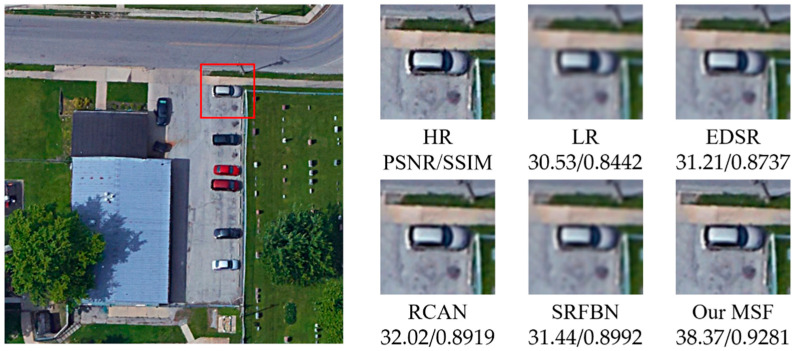
Qualitative comparison between different SR models performance on vehicle instance-class.

**Figure 6:**
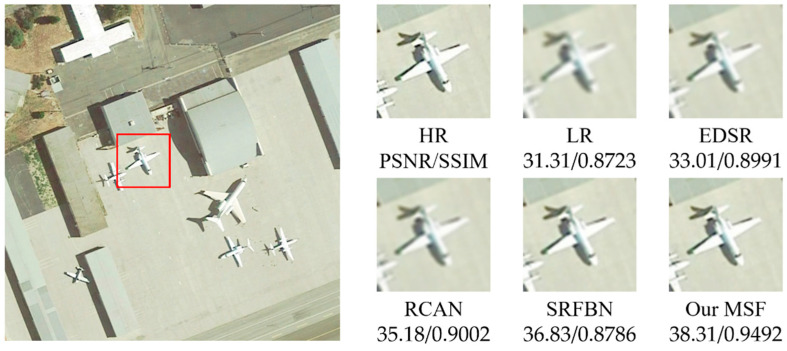
Qualitative comparison between different SR models performance on plane instance-class.

**Figure 7:**
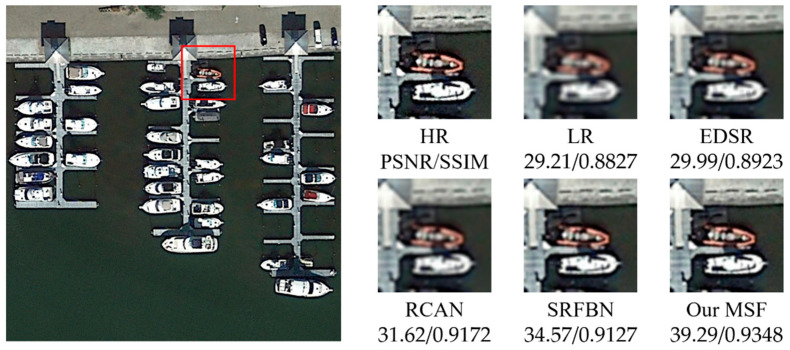
Qualitative comparison between different SR models performance on ship instance-class.

**Figure 8:**
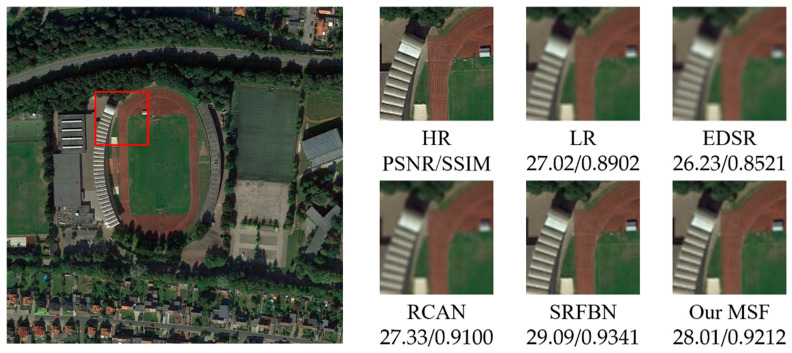
Qualitative comparison between different SR models performance on stadium instance-class.

**Figure 9:**
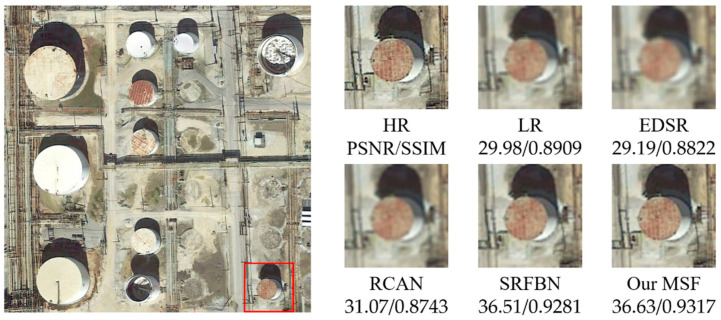
Qualitative comparison between different SR models performance on storage tank instance-class.

Figure 5, Figure 6, Figure 7, Figure 8 and Figure 9 show the qualitative comparison results between our MSF and other SotA SR algorithms when trained and tested on data set over five categories. As shown above, the super-resolved result of our MSF has clearer edges and higher contrast than other SR models. It should be noted that all test images (except the stadium class) contain instances which repeatedly appear with diverse size and orientation. This internal recurrent information provides strong edge and texture predictive power through using unsupervised learning strategy, which cannot be exploited by supervised SR models. For stadium class, aided by the feedback blocks with feedback connection, the ability of SRFBN to super resolve the regular geometric shapes in the test piece slightly excels our MSF, showing clearer lines on the ground and boundaries of the stadium. However, MSF still achieves a comparable result. Considering the SR models for remote sensing images usually pay more attention to the complex and diverse edges and texture of instance objects rather than simple and regular ones, we believe the effectiveness of our MSF in the remote sensing field.

#### 3.2.2. The Influence of Different Scaling Factors

In this part, our purpose is to compare the performance of MSF and other SR algorithms on the data with different scaling factors. We adjust the scaling factor (2,3,4) in the bicubic downsampling step. Theoretically, with a larger scaling factor, the downsampled piece loses more texture information and details, making the framework more struggled to capture the distribution of the degradation model. To obtain an unbiased result, all SR methods test on the new selected data set (class: plane).

The result in Table 2 shows that all SR algorithms obtain remarkably lower scores of PSNR/SSIM with the increase of the scaling factor, as we predicted before the experiment. However, our MSF still outperforms other networks, mostly because the unsupervised module in our framework fully utilizes the internal information recurred in the patch.

Additionally, we hypothesize the dataset expanded by various scaling factors would contribute to performance promotion for MSF, but the experiment shows that this data augmentation method, in fact, confuses the network by decreasing the correspondence between LR-HR pairs and thus weakens the performance of our network.

#### 3.2.3. Ablation Study

The purpose of this section is to demonstrate the importance of three modules in our framework. To do this, we maintain the architecture of the foundation network and ablate or remain the modules in the framework to generate new frameworks. Moreover, to verify the effectiveness of our architecture, we add frameworks with full input (360 pieces) as exclusive input data for CCM or MLM. It should be noted that the ULM is an unsupervised network that only needs the test set. We train six frameworks in parallel: complete MSF (with 200 pieces as train data for CCM; 160 pieces for MLM), CCM (with 360 pieces as train data), MLM+ULM(with 160 pieces as train data for MLM), MLM+ULM(with 360 pieces as train set for MLM, 36 Gaussian blur kernels in MLM), ULM(with only test images), and CCM(360)+ULM(with 360 pieces as train data for CCM). All frameworks test on a new generated test set (class: ship)

Table 3 presents the results of six frameworks. In general, complete MSF remarkably outperforms other incomplete frameworks. What is more, it is shown that the frameworks adopted MLM are noticeably superior to those without MLM, which demonstrates the significance of the multi-task learning strategy. Moreover, the result that a single application of ULM achieves a similar PSNR score with CCM (360) and obviously higher SSIM score than CCM (360) verifies our concern that the recurrent information in the single test image provides strong predictive power for SR procedure.

In detail, we can see that MLM(360) + ULM framework achieves the second-best performance, considerably superior to MSF(160) + ULM, showing more blur kernels that indeed promote the performance of the framework (36 kernels versus 16 kernels). Nevertheless, the difference of 1.84 dB from MSF verifies the effectiveness of the CCM. Besides, the running time of MLM(360) + ULM is 13 hours longer than that of MSF, which also demonstrates the accelerating function of CCM. The single CCM(360), whose architecture is a typical supervised SR network, achieves only 31.31 dB and 0.8464, inferior to ULM with only one test image. This result mainly springs from the fact that the nature of SR model for real scenarios is a nonconvex optimization, and the optimized parameter of a network solely learning the large-scale external dataset shows performance drop when testing on the images with unknown blur kernel.

**Figure 10:**
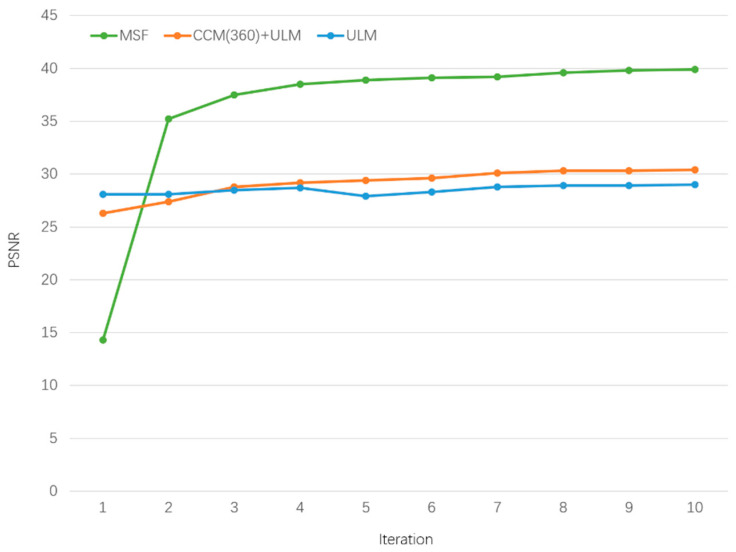
The relationship between PSNR score and the number of iterations in three unsupervised learning modules.

To investigate the potential of the unsupervised learning strategy in the SR field, we record the relationship between PSNR score and the number of iterations in the test phase of frameworks that contains unsupervised module (i.e., MSF(360), ULM, and CCM(360)+ULM). Astonishingly, as shown in Figure 10, the single ULM achieves the best performance when there is only one iteration. On the contrary, the ULM initialized with the parameters from CCM does not show better performance, which proves that simply combining the supervised learning module with the unsupervised learning module will not enhance the framework’s ability to tackle the test image with an unknown blur kernel. In other words, the MLM wisely exploits the external prior to reinforce the performance of the ULM. The result that our MSF obtains the least score in the first iteration is reasonable because the purpose of the MLM is to find a transferable and sensitive point instead of a local optimum. Obviously, when the second iteration ends, our MSF has remarkable edges over the other two frameworks. In addition, our MSF coverages in about 10 iterations whereas the CCM+ULM framework needs about 200 iterations and ULM needs approximately 2500 iterations.

To further study the influence of the number of subordinate networks in MLM on the SR performance, we design a comparison test. Five MSFs are trained in parallel and the amount of data remains the same, 200 image pairs in CCM and 160 image pairs in MLM. The difference among five frameworks is the number of subordinate networks in MLM (that is, the number of Gaussian blur kernels generated in MLM): 4, 8, 16, 32, 80, respectively. Therefore, the amounts of data that corresponds to each subordinate network are 40, 20, 10, 5, 2, respectively.

**Figure 11:**
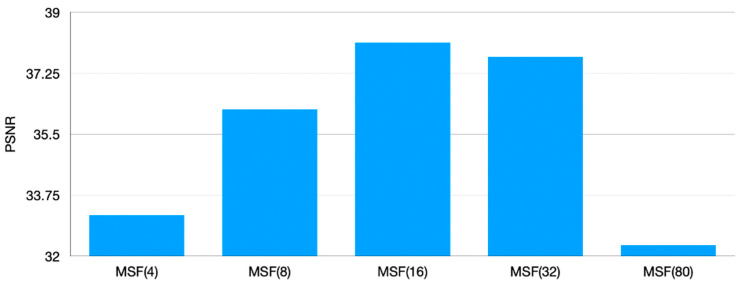
Comparison among MSF with different number of subordinate networks.

Figure 11 shows that the MSF performance is positively correlated with the number of the subordinate networks in MLM in the range between 4 and 16. However, the performance of MSF(32) has a decreasing tendency and MSF(80) shows a dramatical performance drop. It may be attributed to the small amount of data for each subordinate network. Hence, to further verify our speculation, we conduct a comparison test, in which three MSF frameworks with 16, 32, 80 subordinate networks in MLM are trained in parallel. 160 image pairs are randomly generated into 32 and 80 groups with some data being repeatedly selected, aiming to ensure that each subordinate network corresponds to 10 image pairs, which are same as MSF(16).

**Figure 12:**
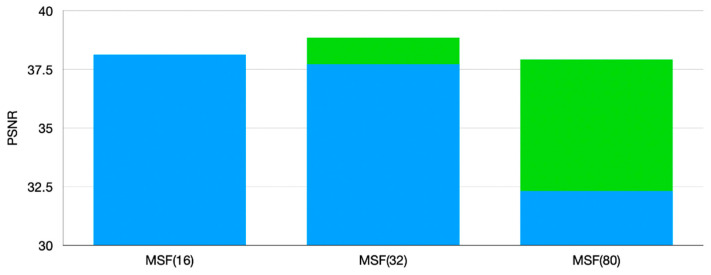
Comparison among MSF with different number of subordinate networks after data augmentation step; the green part is the increase obtained by data augmentation.

**Figure 13:**
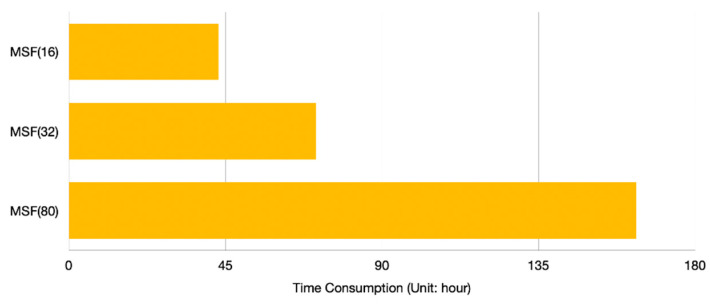
Time consumption of different MSF.

As one can see in the Figure 12, the data augmentation method that increases the training samples in each subordinate network can restore the effectiveness of MSF damaged by the small amount of training data. Nevertheless, the performance of MSF(80) is still inferior to those of MSF(16) and MSF(32), which proves that the number of blur kernels in is not always positively correlated to the performance of the proposed SR framework. Besides, it can be observed in Figure 13 that the time consumptions of MSF(32) and MSF(80) are obviously higher than that of MSF(16). Moreover, the MSF with more blur kernels in MLM frequently encounters gradient vanishing problems and gradient exploding problems. As a result, we set 16 subordinate networks in MLM as default.

#### 3.2.4. Super Resolution for Real Remote Sensing Images

In addition to the previous experiments on synthetic LR-HR image pairs to quantitively evaluate the performance of our proposed MSF, the experiment on instance-pieces in DIOR as direct input to our framework is also conducted to test the effectiveness of MSF. Without groud-truth HR images, only visual results are provided to show the comparison between the original image and predicted SR output. In the experiment, different spatial-resolution instance-pieces are taken as input to MSF.

**Figure 14:**
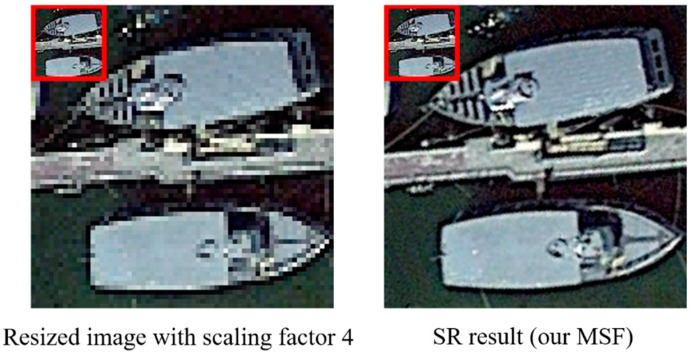
Qualitative comparison between instance-piece from DIOR and SR output of MSF.

The visual comparison is shown in Figure 14. We can see that after resizing step with scaling factor 4, the edge of the ships has mosaic effect and becomes serrated. The SR result from our MSF has smooth edge and high contrast, achieving better visual pleasing results. It is because the ships in the original image have relatively simple edges and details, and prior information extracted by CCM and recurred information captured by ULM enhance the predictive power of the SR model. However, our SR result show some limitation. First, although detail information is added, the body of both ships still has some objects unrecognizable. Second, some linear artifacts appear in the body of the upper ship.

## 4. Discussion

The reconstruction process of the super resolution algorithms is based on the degradation model, as shown in Equation (1). However, most of the current SotA SR algorithms usually assume the blur kernel in degradation is predefined (e.g., bicubic downsampling). Hence these SR algorithms usually suffer a performance drop when tested on remote sensing images because the blur kernel in the real world is usually a Gaussian blur kernel with unknown parameters. Therefore, a SR algorithm that can tackle unknown Gaussian blur kernel is needed for remote sensing images.

In this study, we introduce the multi-task learning strategy, through which our framework learns the general features among SR reconstruction process with different Gaussian blur kernel. In addition, the external information and internal recurred information are employed to provide useful high-frequency information and details to SR result and accelerate the training process of the multi-task learning module.

Through the comparison experiment, our MSF outperform other state-of-the-art SR algorithms with a large gap on the vehicle, ship, and plane classes. The distinctive performance of our MSF springs from its ability to tackle various blur kernels and to utilize the recurrent information to reason the estimated blur kernel in the test image. On the stadium and storagetank classes, our MSF still achieves comparable result to SRFBN, remarkably superior to EDSR and RCAN. In particular, there is a tendency that the results on the stadium scenario are remarkably inferior to other scenarios. It can be explained by the data preparation step that the size of the entire stadium instance is usually larger than the size of the input tensor to the framework, so the features learned by these SR algorithms lack visual correlation. What is more, all SR algorithms achieve relatively high SSIM scores. This is perhaps due to the small size and simple architecture of remote sensing instances. Normally these SotA SR algorithms on DIV2K dataset obtain SSIM scores between 0.7 to 0.8.

To further verify the MSF performance with different scaling factors, we conduct comparison experiments with other SotA methods with three scaling factors. Our MSF shows the best result for all scaling factors, which indicates the ability of MSF to restore the high frequency information and texture in remote sensing instances is superior to other SotA SR methods. Since all algorithms are tested on the same dataset, we speculate the remarkable effectiveness of our MSF comes more from the multi-task learning strategy than from the external and internal information. To verify this speculation and quantify the significance of three modules, we conduct ablation study. The result demonstrates that framework with MLM module can dramatically promote the performance of SR methods in reconstruction process. Conversely, the frameworks that solely employ external information and internal information show only slightly improvement compared with the LR image.

Nevertheless, the result that the performance of MSF outperforms the combination of MLM and ULM proves that the number of the subordinate networks in MLM does not always benefit the SR framework. To further evaluate the relationship between the number of the subordinate networks and the effectiveness of our proposed method, we compare the performance and time consumption of MSF with different subordinate networks in MLM. The result shows that, although adding more subordinate networks to the framework in the first place promotes the MSF ability to tackle the LR image with unknown Gaussian blur kernel, too much subordinate networks negatively correlate to the performance. Besides, the time consumption continuously increases with more blur kernels in MLM. Therefore, it is a tradeoff between the performance and computational resource.

In addition to the above experiments on synthetic test images, we also conduct experiments on the real remote sensing instances to test the effectiveness of our MSF. With no ground truth image, the visualization result shows that our MSF can provide high frequency information to low resolution remote sensing instance, making the edge of the instance clearer and the contrast more sharpening. However, it is worth noting that, some artifacts appeared in the instance. Moreover, though with better visual pleasing instance, some objects in the instances cannot be classified, so how the features derived from the SR process can facilitate the classification and object detection tasks needs more research.

## 5. Conclusions

In this paper, we have proposed a new framework to tackle the super-resolution problem in remote sensing images by exploiting a multi-task learning strategy and an unsupervised learning strategy. In order to fully learn the implicitly representative features among degradation models with different blur kernels, we introduced the multi-task learning module, in which each subordinate task corresponded to a randomly generated Gaussian blur kernel and the main network was optimized by the combined results from the subordinate networks. Coupled with the class-feature capture module, which trained on the large-scale external dataset to learn the class-relevant information, and the unsupervised learning module, which estimated the blur kernel within the test image by utilizing the recurrent information in the text image, our framework overcame the shortcoming of multi-task learning strategy and efficiently obtained remarkable results. Various experiments on the remote sensing dataset DIOR, either with several SotA SR algorithms or with different scaling factors, demonstrated the effectiveness of our framework when facing the LR images downsampled by a randomly generated Gaussian blur kernel. On the benchmark with scaling factor 2, our proposed framework remarkably outperformed other SR networks, increasing resolution by at least 19%, 13.6%, 4%, and 0.3% in vehicle, ship, plane, and storage-tank categories, respectively. Besides, the proposed framework still obtained best performance when facing the super-resolution task with large scaling factors, diminishing the effect of information loss and super resolving the low-resolution image with sharp contrast and clear edge. Moreover, we conducted an ablation study to compare six frameworks, showing the advantage of our proposed MSF and the potential of the unsupervised super-resolution model on the remote sensed imagery. Finally, we tested the performance of the proposed framework MSF on the real-world remote sensing image without ground truth. The result proved the effectiveness of our model’s ability to super resolve the low-level vision instance while reminding us of the possibility of adding incorrect information when facing instances with complex structure and diverse details.

However, there are improvements that may benefit our work, such as the more efficient class-feature capture module, more lightweight strategy for multi-task learning module, more practical loss function, and more precise blur kernel estimation. We leave these for future work.

## Figures and Tables

**Table 1:** The average PSNR/SSIM results of SR models on five categories of instances with a new randomly generated Gaussian blur kernel (scaling factor 2).

Instance	Bicubic	EDSR	RCAN	SRFBN	MSF
Vehicle	30.53/0.8442	31.21/0.8737	32.02/0.8919	31.44/0.8992	38.37/0.9281
Ship	29.21/0.8827	29.99/0.8923	31.62/0.9172	34.57/0.9127	39.29/0.9348
Plane	31.31/0.8723	33.01/0.8991	35.18/0.9002	36.83/0.8786	38.31/0.9492
Stadium	27.02/0.8902	26.23/0.8521	27.33/0.9100	29.09/0.9341	28.01/0.9212
Storage tank	29.98/0.8909	29.19/0.8822	31.07/0.8743	36.51/0.9281	36.63/0.9317

**Table 2:** The average PSNR/SSIM results on dataset with different scaling factors.

Scaling Factor	Bicubic	EDSR	RCAN	SRFBN	MSF
×2	30.13/0.8003	33.44/0.8464	34.16/0.8821	36.22/0.8896	39.91/0.9429
×3	26.10/0.7112	27.69/0.8102	29.91/0.8434	30.01/0.8632	34.21/0.9102
×4	24.19/0.6928	23.52/0.7676	24.32/0.8002	28.32/0.8116	29.32/0.9008

**Table 3:** The average PSNR/SSIM results on six frameworks.

MSF(360)	CCM(360)	MLM(160) + ULM	MLM(360) + ULM	ULM	CCM(360) + ULM
39.77/0.9378	31.31/0.8464	33.29/0.8894	37.93/0.9369	31.22/0.8899	32.13/0.8852

## Data Availability

Data available in a publicly accessible repository that does not issue DOIs. Publicly available datasets were analyzed in this study. This data can be found here (https://pan.baidu.com/share/init?surl=w8iq2WvgXORb3ZEGtmRGOw passcode: 554e; accessed date: 2 March 2021).

## Abbreviations

SR	super-resolution
HR	high-resolution
LR	low-resolution
SISR	single-image super-resolution
MISR	multi-image super-resolution
MSF	multiple-blur-kernel super-resolution framework
CCM	class-feature capture module
MLM	multiple-blur-kernel learning module
ULM	unsupervised learning module
SotA	state-of-the-art
